# Abnormal CYP11A1 gene expression induces excessive autophagy, contributing to the pathogenesis of preeclampsia

**DOI:** 10.18632/oncotarget.21158

**Published:** 2017-09-22

**Authors:** Tianying Pan, Guolin He, Meng Chen, Chenyi Bao, Yan Chen, Guangyu Liu, Mi Zhou, Shuying Li, Wenming Xu, Xinghui Liu

**Affiliations:** ^1^ Department of Obstetrics and Gynecology, West China Second University Hospital, Sichuan University, Chengdu 610041, China; ^2^ Joint Laboratory of Reproductive Medicine, Sichuan University-The Chinese University of Hong Kong, Chengdu 610041, China; ^3^ Key Laboratory of Birth Defects and Related Diseases of Women and Children, Ministry of Education, West China Second University Hospital, Sichuan University, Chengdu 610041, China

**Keywords:** CYP11A1, autophagy, preeclampsia, testosterone, androgen receptor

## Abstract

**Objective:**

In this study, we investigated the exact mechanism by which excessive CYP11A1 expression impairs the placentation process and whether this causes preeclampsia (PE) in an *in vivo* model.

**Setting and Design:**

In order to study CYP11A1 overexpression, BeWo cells were transfected with CYP11A1. Pregnenolone, progesterone, and testosterone levels were measured by enzyme linked immunosorbent assays, and levels of autophagy markers were quantified by western blotting and immunofluorescence. Trophoblastic cell invasion was assessed using transwell assays; BeWo cells were treated with testosterone and an androgen receptor (AR) inhibitor (flutamide) to elucidate the invasion mechanism. An adenovirus overexpression rat model was established to investigate CYP11A1 overexpression *in vivo* and the phenotype was examined. Furthermore, human placenta samples (*n* = 24) were used to determine whether PE patient placentas showed altered CYP11A1 and autophagy marker expression.

**Results:**

BeWo cells overexpressing CYP11A1 had significantly increased levels of pregnenolone, progesterone, and testosterone. Additionally, the expression levels of autophagy markers in CYP11A1-overexpressing BeWo cells were significantly increased. Trophoblast invasion was significantly reduced in CYP11A1-overexpressing cells as well as in cells treated with high testosterone. This reduction could be significantly rescued when cells were pretreated with flutamide. Overexpression of CYP11A1 in rat pregnancies led to PE-like symptoms and an over-activation of the AR-mediated pathway in the placenta. Elevated expression of CYP11A1 and autophagy markers could also be detected in PE placenta samples.

**Conclusions:**

These results suggest that abnormally high expression of CYP11A1 induces trophoblast autophagy and inhibits trophoblastic invasion, which is associated with the etiology of PE.

## INTRODUCTION

Preeclampsia (PE) is a pregnancy-specific hypertensive disease with multisystem involvement that is primarily defined by the occurrence of new-onset hypertension with new-onset proteinuria [1]. Hypertensive disorder occurs in 5–10% of all pregnancies, and PE is identified in 3.9% of all pregnancies. PE is a leading cause of maternal and perinatal morbidity and mortality; an estimated 50,000–60,000 PE-related deaths occur per year worldwide [2]. Although the pathogenesis of PE is still not fully understood, impaired trophoblastic invasion into maternal spiral arteries, which causes poor vascular remodeling leading to endothelial and placental damage, is of central importance [3].

The Cytochrome P450 Family 11 Subfamily A Member 1 (CYP11A1) gene encodes a cholesterol side chain cleavage enzyme (cytochrome P450 cholesterol side-chain cleavage, P450scc) that catalyzes the first step of steroidogenesis, where cholesterol is converted to pregnenolone (a precursor for all other steroid hormones) [4]. During pregnancy, the placenta synthesizes a large number of steroid hormones that are closely associated with the beginning and maintenance of pregnancy, fetal development, and delivery [5]. CYP11A1 is expressed in the placenta and is responsible for placenta-derived hormone synthesis, including the hormones progesterone and testosterone. Progesterone supports pregnancy and prepares the uterine endometrium for embryo implantation during early pregnancy [6]. Plasma levels of testosterone are increased in pathological pregnancies such as PE, and the testosterone levels in preeclamptic women are positively correlated with average systolic blood pressure (SBP) [7]. CYP11A1 is essential for steroid biosynthesis; therefore, abnormal expression of CYP11A1 may affect steroid levels [8]. In our previous study, we found that CYP11A1 gene expression was significantly increased in the placenta during severe PE compared to that in normal pregnancies, as measured by both mRNA and protein levels. Furthermore, CYP11A1 overexpression in a trophoblast cell line induced apoptosis [9]. However, the underlying mechanism by which increased CYP11A1 expression influences steroid levels and PE pathogenesis remained elusive.

Autophagy is a process of self-degradation of cellular components; double-membrane autophagosomes sequester organelles and fuse with lysosomes so that the contents can be digested by lysosomal enzymes [10, 11]. Recent studies have indicated that over-activation of autophagy could promote cellular dysfunction through excessive degradation of essential cellular constituents [12]. Under physiological conditions of low oxygen, autophagy is essential for extravillous trophoblast (EVT) function and invasion, and for vascular remodeling [13]. However, excessive autophagy is also thought to be an important trait in the placenta during PE pathogenesis [14]. Nevertheless, whether the change in expression of pregnancy related hormone levels is related to autophagy remains unclear. We propose that abnormal expression of CYP11A1 could cause altered steroid secretion, which may change autophagy activity and reduce the invasion of EVTs. In this study, we investigated our hypothesis using both *in vitro* and *in vivo* models, as well as human samples. Our results demonstrate a novel molecular pathway where CYP11A1-dependent hormone secretion located in the mitochondrial inner membrane leads to excessive mitophagy (a mitochondria-specific autophagy) that results in compromised (EVT) invasion. Consequently, our study has revealed an important link between hormone synthesis and excessive mitophagy that leads to reduced EVT invasion during PE pathogenesis.

## RESULTS

### Overexpression of CYP11A1 increased pregnenolone-mediated hormone secretion and inhibited invasion

**Figure 1:**
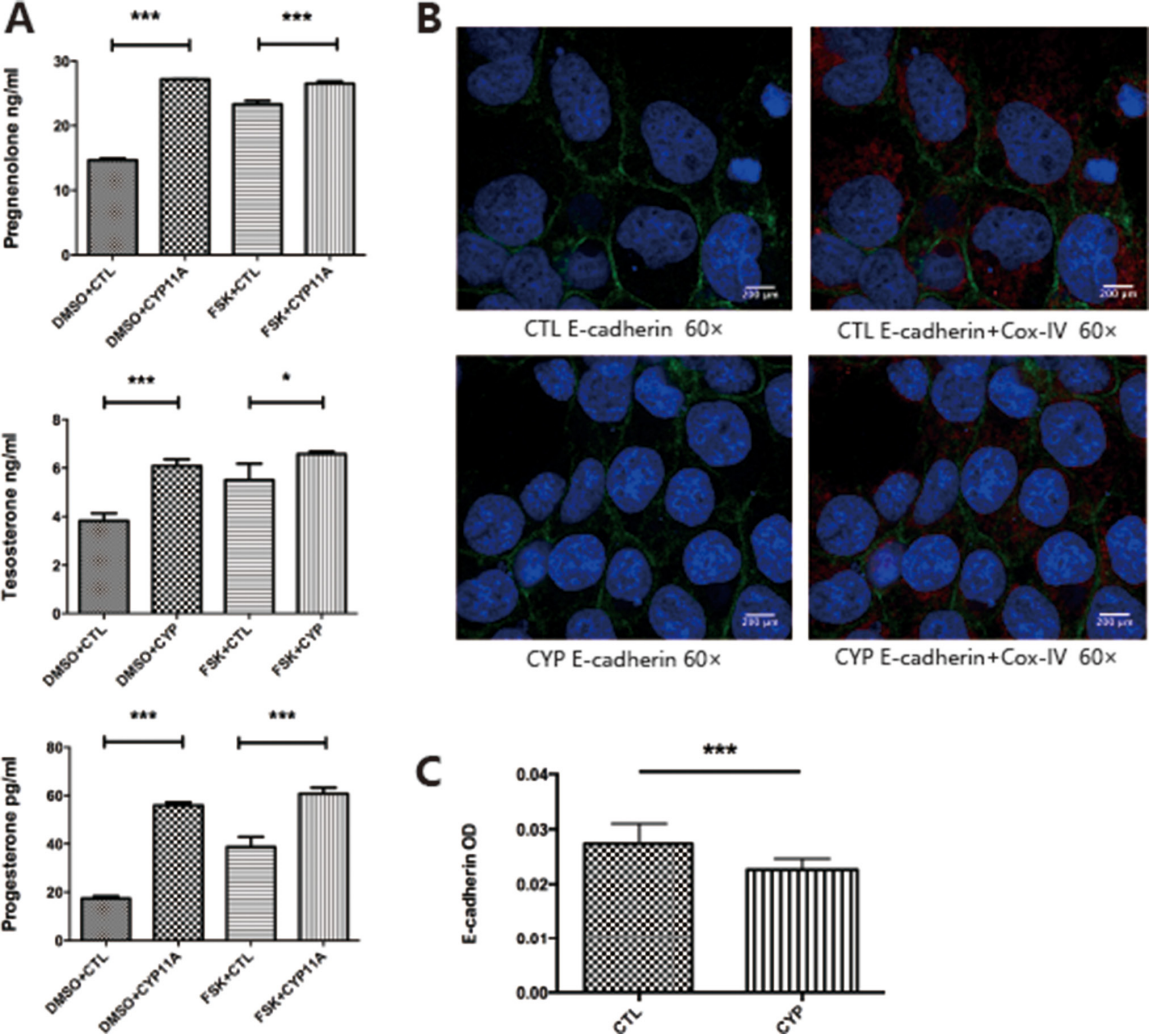
Overexpression of CYP11A1 induces androgen production and syncytiotrophoblast formation in BeWo cell line (**A**) Overexpression of CYP11A1 and forskolin (10 μM) treatment increase pregnenolone, progesterone and testosterone secretion in BeWo cell line. (**B**, **C**) E-cadherin staining show that overexpression of CYP11A1 could reduce E-cadherin staining intensity, indicating syncytiotrophoblast formation in BeWo cell line(60×). Cox-IV was used as mitochondrial marker. * means *P* < 0.05, ** means *P* < 0.01, *** means *P* < 0.001. FSK = foskolin, 3.1 = control plasmid (pcDNA-3.1), CYP = CYP11A1 over expression plasmids (pcDNA-3.1-CYP11A1).

The BeWo cell line was used for our studies, as the BeWo cell line is a well-established cell model used to mimic primary trophoblast hormone secretion. We overexpressed CYP11A1 to determine whether over-activated CYP11A1 could alter steroid secretion. Hormones downstream of CYP11A1, including pregnenolone, progesterone, and testosterone, were measured by ELISA. We observed that overexpression of CYP11A1 increased the secretion of these key hormones (Figure 1A). E-cadherin staining disruption is a marker of syncytiotrophoblast formation. We found that overexpression of CYP11A1 induced syncytiotrophoblast formation in the BeWo cell line (Figure 1B, 1C). Therefore, this *in vitro* overexpression system could be used to investigate the effect of abnormal expression of CYP11A1 on trophoblast cell behavior.

### CYP11A1 overexpression and androgen treatment induced mitophagy in trophoblast cells

**Figure 2:**
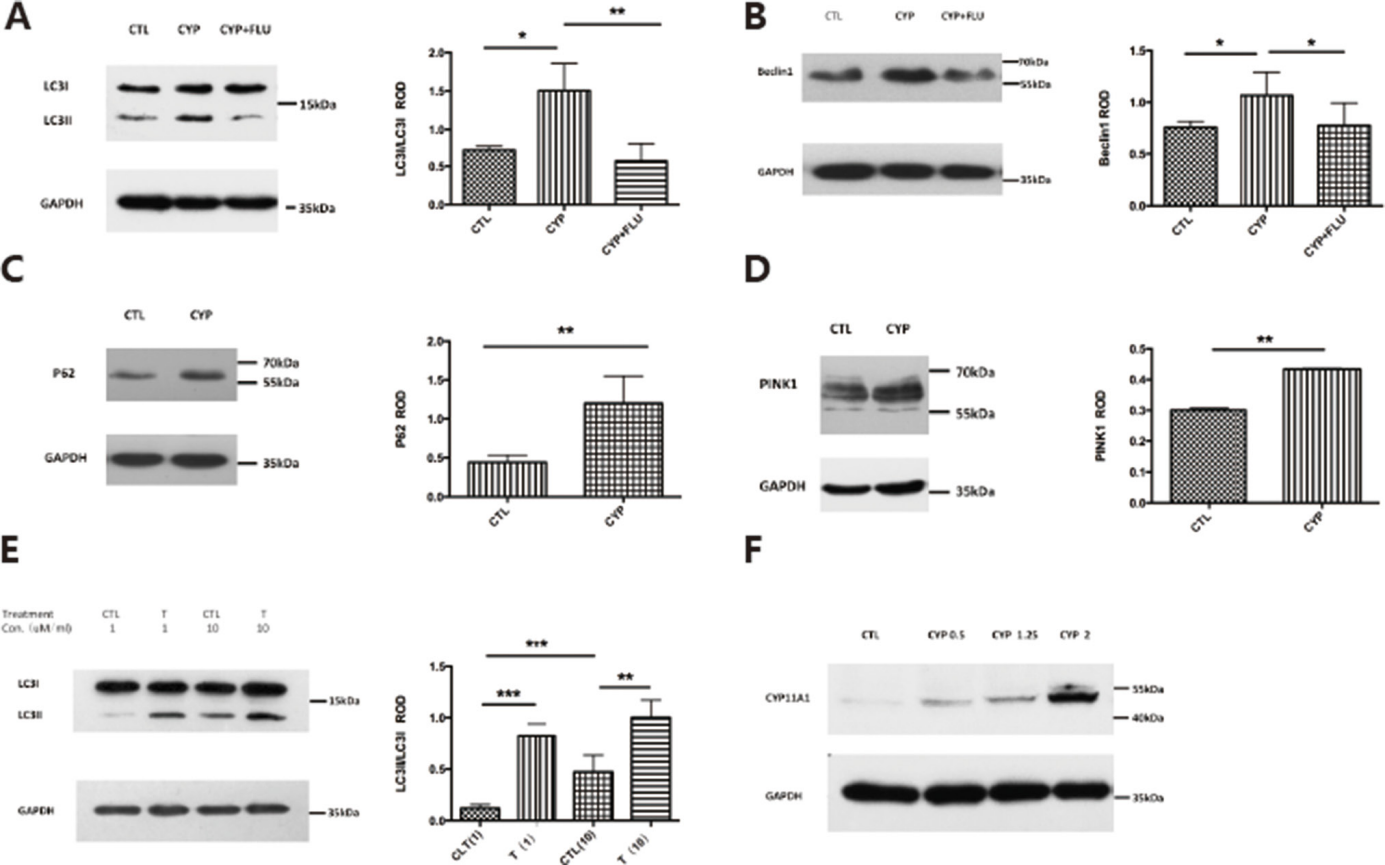
CYP11A1 overexpression and androgen could induce autophagy in BeWo cell line (**A**) Western blot result shows that CYP11A1 overexpression induces LC-3II expression, while flutamide (10 μM) could inhibit the LC-3II expression. (**B**) Beclin 1 expression would also be induced after CYP11A1 overexpression, which could be blocked by androgen receptor (AR) inhibitor, flutamide. (**C**, **D**) P62 and Pink1 expression would also be induced by CYP11A1 expression. (**E**) Testosterone could induce LC-3II expression dose-dependently after 48 h treatment. (**F**) Overexpression of increasing amount (0.5 μg,1 μg,2 μg CYP11A1) plasmid induce CYP11A1 protein expression * means *P* < 0.05, ** means *P* < 0.01, *** means *P* < 0.001.

Previous studies have shown that autophagy levels are increased in early-onset PE. To investigate whether abnormal expression of CYP11A1 could increase autophagy, western blotting was used to measure the expression of autophagy biomarkers. LC3-II is a widely used biomarker to determine autophagy induction, and our results showed that LC3-II expression was significantly increased in BeWo cells transfected with CYP11A1 (Figure 2A). Beclin1 is another marker for autophagy, which was also found to be increased in CYP11A1-transfected cells, indicating that autophagy was induced after CYP11A1 overexpression (Figure 2B). Interestingly, P62 and PINK-1 expression levels were also induced by CYP11A1 overexpression, while ATG5 and VDAC1 expression levels were suppressed, indicating that CYP11A1 could specifically affect LC3-II and P62 expression during autophagy activation (Figure 2C, 2D and Supplementary Figure 1A, 1B). Interestingly, testosterone treatment also increased autophagy marker expression (Figure 2E), indicating that CYP11A1-activated testosterone could specifically activate autophagy in trophoblasts. The effect of CYP11A1 overexpression was confirmed via western blotting (Figure 2F); increasing the amount of CYP11A1 plasmid transfection led to a dose-dependent expression response of autophagy markers.

**Figure 3:**
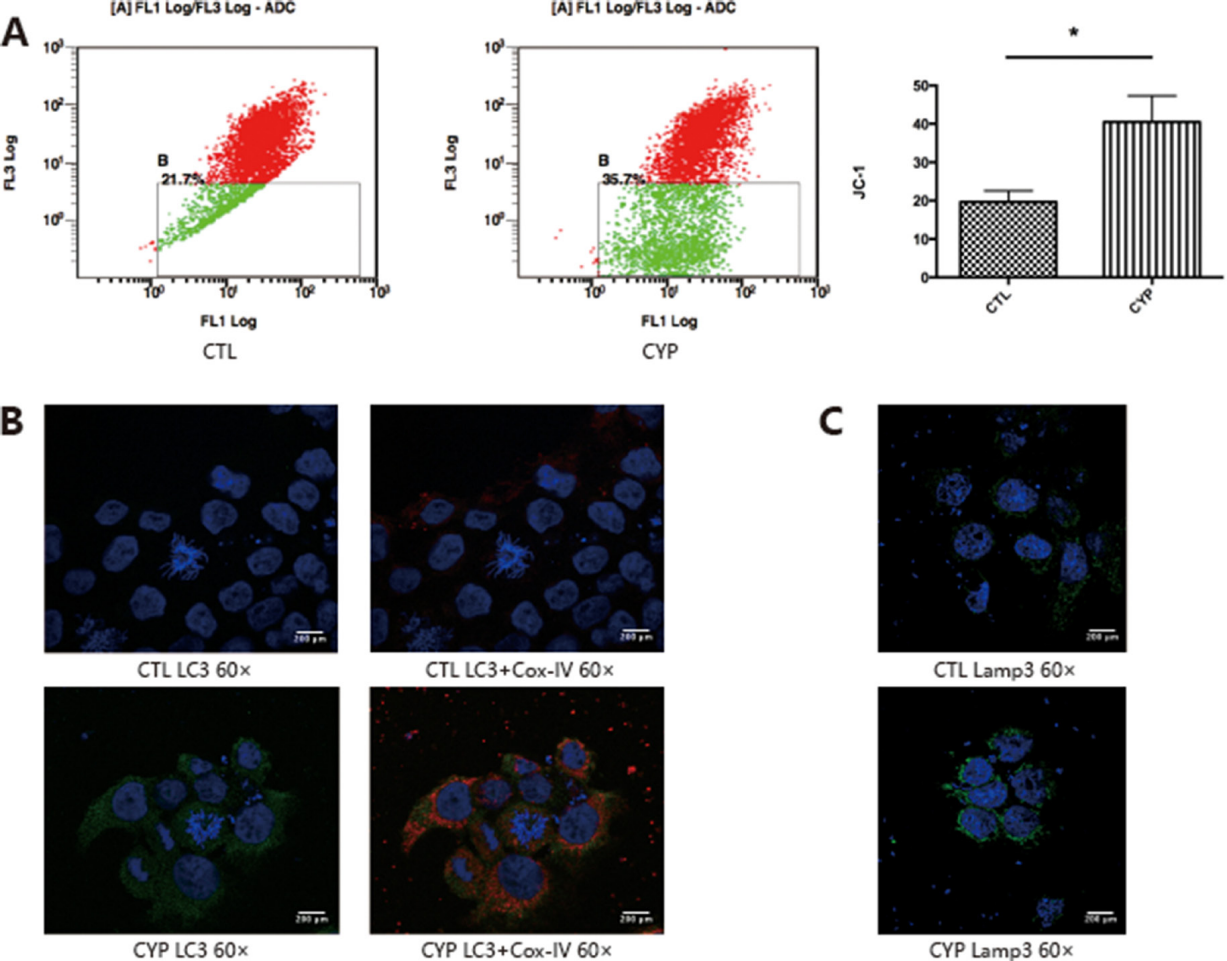
CYP11A1 overexpression lead to mitochondrial member potential depolarization and mitophagy in BeWo cell line (**A**) Flow cytometry analysis of JC-1 staining indicate that overexpression of CYP11A1 induce member potential depolarization in BeWo cell line. (**B**) Immunofluorescence staining shows that overexpression of CYP11A1 induces mitochondrial localization of LC-3. Cox-IV was used as mitochondrial marker. Increased colocalization of LC-3 (green) and Cox-IV (red) was shown after CYP11A1 overexpression. (**C**) Immunofluorescence staining show that overexpression of CYP11A1 induces Lamp3 expression. Colocalization of LC-3 (green) and Cox-IV (red) was shown in the right panel. * means *P* < 0.05.

Over-activated autophagy could lead to oxidative stress and mitochondria play a critical role in autophagy. Since CYP11A1 is expressed in the mitochondrial inner membrane, we investigated whether overexpression of CYP11A1 could lead to mitochondrial membrane potential depolarization. JC-1 staining demonstrated that overexpression of CYP11A1 can cause mitochondrial dysfunction (Figure 3A), as indicated by the increased permeability of the mitochondria. Since CYP11A1 is a mitochondrial located protein, immunofluorescence of autophagy and lysosome markers, LC-3II and Lamp3, was performed along with co-staining for the mitochondrial protein Cox-IV to confirm their expression and localization after CYP11A1 overexpression. Increased colonization of the mitochondrial marker Cox-IV and LC-3A/B was observed upon CYP11A1 overexpression showing that mitophagy, a selective degradation of mitochondria, was significantly increased (Figure 3B). CYP11A1 overexpression also led to Lamp3 overexpression (Figure 3C), indicating that CY11A1 may significantly activate mitophagy and lysosome-mediated degradation in trophoblasts.

### Overexpression of CYP11A1 inhibited trophoblast invasion through regulation of MMP2

**Figure 4:**
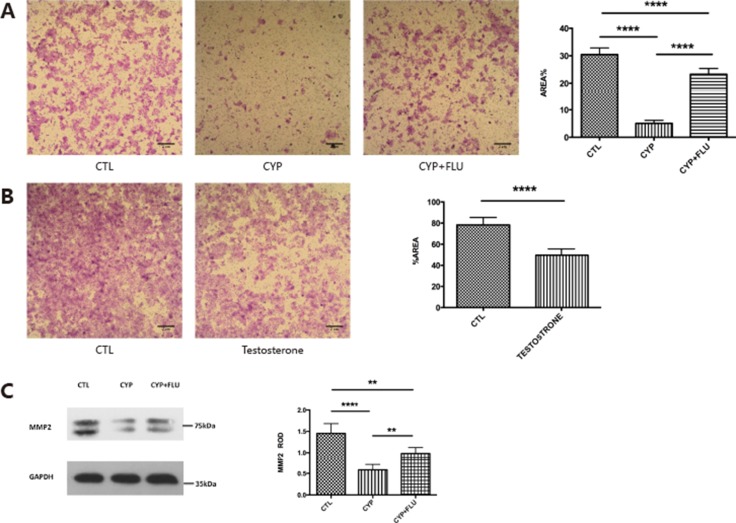
CYP11A1 overexpression compromise trophoblast invasion and inhibit MMP-2 expression (**A**) The picture shows that trophoblast invasion could be inhibited by CYP11A1 overexpression, which could be rescued by flutamide treatment. (**B**) Testosterone treatment could inhibit trophoblast invasion. (**C**) Western blot result of MMP-2 shows that overexpression of CYP11A1 could inhibit MMP-2 expression, which could rescued by androgen receptor (AR) inhibitor, flutamide. The statistic result is shown in the right panel. * means *P* < 0.05, ** means *P* < 0.01, *** means *P* < 0.001,****means *P* < 0.0001.

It has been shown that testosterone changes during pregnancy can affect trophoblast invasion; therefore, we examined whether CYP11A1 overexpression leads to changes in trophoblast behavior. A trophoblast invasion assay was performed on BeWo cells transfected with CYP11A1. The percentage of BeWo cells that had migrated following overexpression of CYP11A1 was significantly reduced compared to that of untreated BeWo cells (Figure 4A). Since CYP11A1 increased testosterone levels in our overexpression experiment and testosterone exerts its major function through the AR-mediated pathway in trophoblast cells, we used flutamide, an AR inhibitor, to pretreat cells overexpressing CYP11A1, and found that AR inhibition significantly rescued cell invasion compared to that in the non-treated CYP11A1-overexpressing cells (Figure 4A). This indicates that the AR is a critical regulator of CYP11A1-mediated trophoblast invasion. Trophoblast invasion was also significantly reduced upon testosterone treatment (Figure 4B), indicating that both CYP11A1 and testosterone could reduce trophoblast invasion through the AR-mediated pathway. Matrix metallopeptidase proteins, such as MMP2, are major players mediating trophoblast invasion. Therefore, we examined whether overexpression of CYP11A1 affected MMP2 expression. Western blotting showed that overexpression of CYP11A1 significantly compromised MMP2 expression, and that this could be rescued by inhibiting the AR (Figure 4C). Our results indicate that abnormal CYP11A1 expression could inhibit BeWo cell invasion via regulation of MMP2 expression, and that the AR plays a central role in the regulatory process.

### CYP11A1 overexpression led to PE-like symptoms and over-activated autophagy in trophoblasts *in vivo*

**Figure 5:**
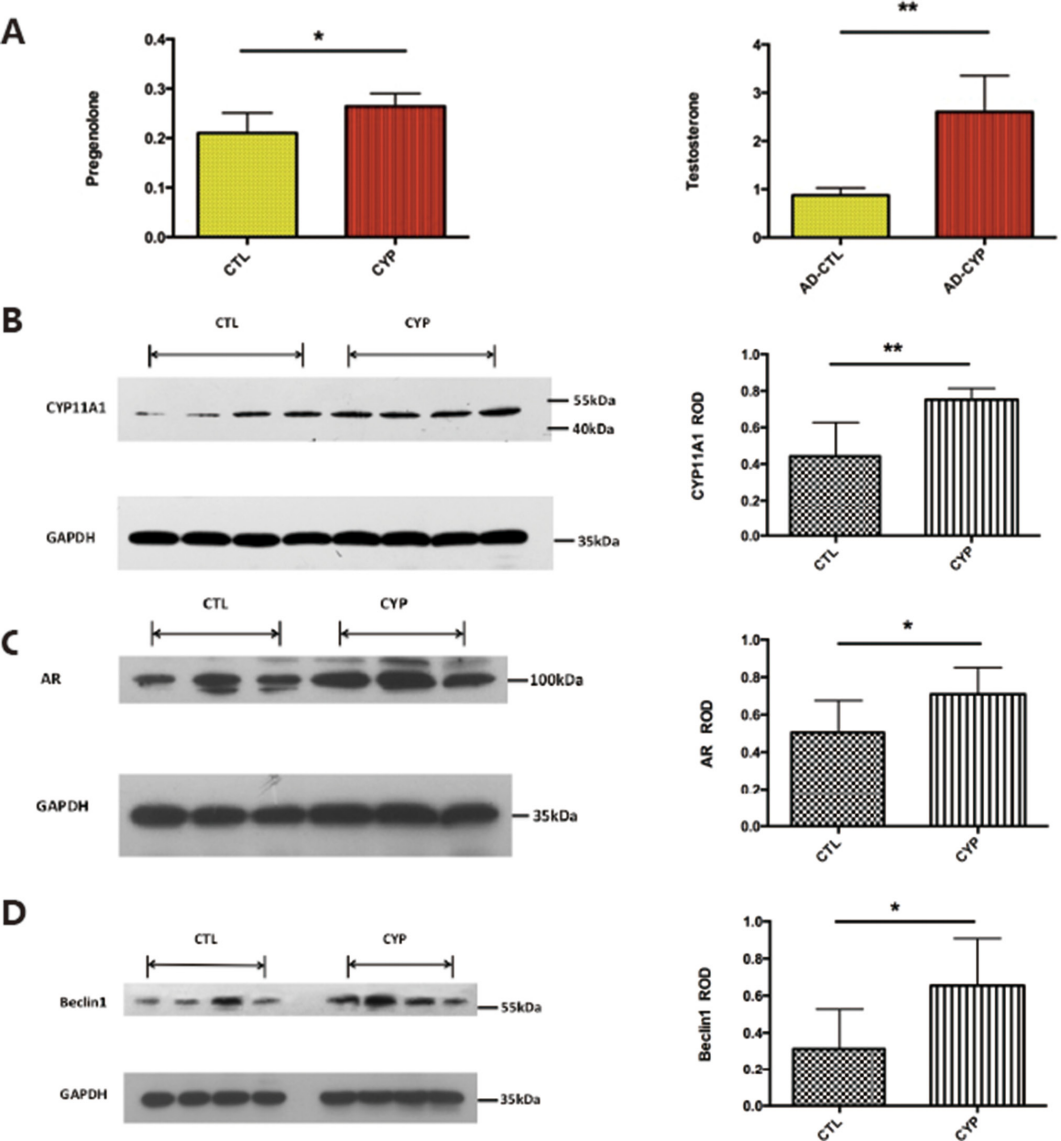
Adenovirus induced CYP11A1 expression in pregnant rats induce androgen receptor (AR) mediated pathway (**A**) ELISA measurement result shows that the serum of CYP11A1 overexpression rat significantly increased of pregnenolone and testosterone after adenovirus injection. (**B**) Western blot result shows that the placenta of CYP11A1 overexpressed rat expressed higher CYP11A1. (**C**) Western blot result shows that androgen receptor (AR) expression is significantly increased in CYP11A1 overexpressed rat model compared with controls. (**D**) Beclin1expression in the CYP11A1-overexpressed rat model is significantly higher than the control group. * means *P* < 0.05, ** means *P* < 0.01, *** means *P* < 0.001,****means *P* < 0.0001.

Since our *in vitro* data showed that CYP11A1 overexpression could lead to over-activated autophagy, we next established a rat model to determine whether CYP11A1 overexpression could lead to over-activated autophagy *in vivo*. Pregnant rats were injected with CYP11A1 overexpression adenovirus with a vehicle on GD 8.5. On GD 19.5 testosterone was measured in the plasma using mass spectrometry. Injection of CYP11A1 overexpression virus increased pregnenolone and testosterone levels in the plasma, indicating successful establishment of a CYP11A1-overexpression model (Figure 5A). Furthermore, western blotting of the placenta from CYP11A1 adenovirus injected rats showed significantly increased CYP11A1 expression, indicating that adenovirus injection increased CYP11A1 expression in the placenta (Figure 5B). Furthermore, AR expression was increased in the placentas of the rats overexpressing CYP11A1 (Figure 5C), indicating that CYP11A1 overexpression induced the AR-mediated pathway. The autophagy-related protein Beclin1 was also significantly increased in the rats overexpressing CYP11A1 compared to that in the control (Figure 5D), indicating that CYP11A1 overexpression may increase autophagy activity, potentially leading to PE.

**Figure 6:**
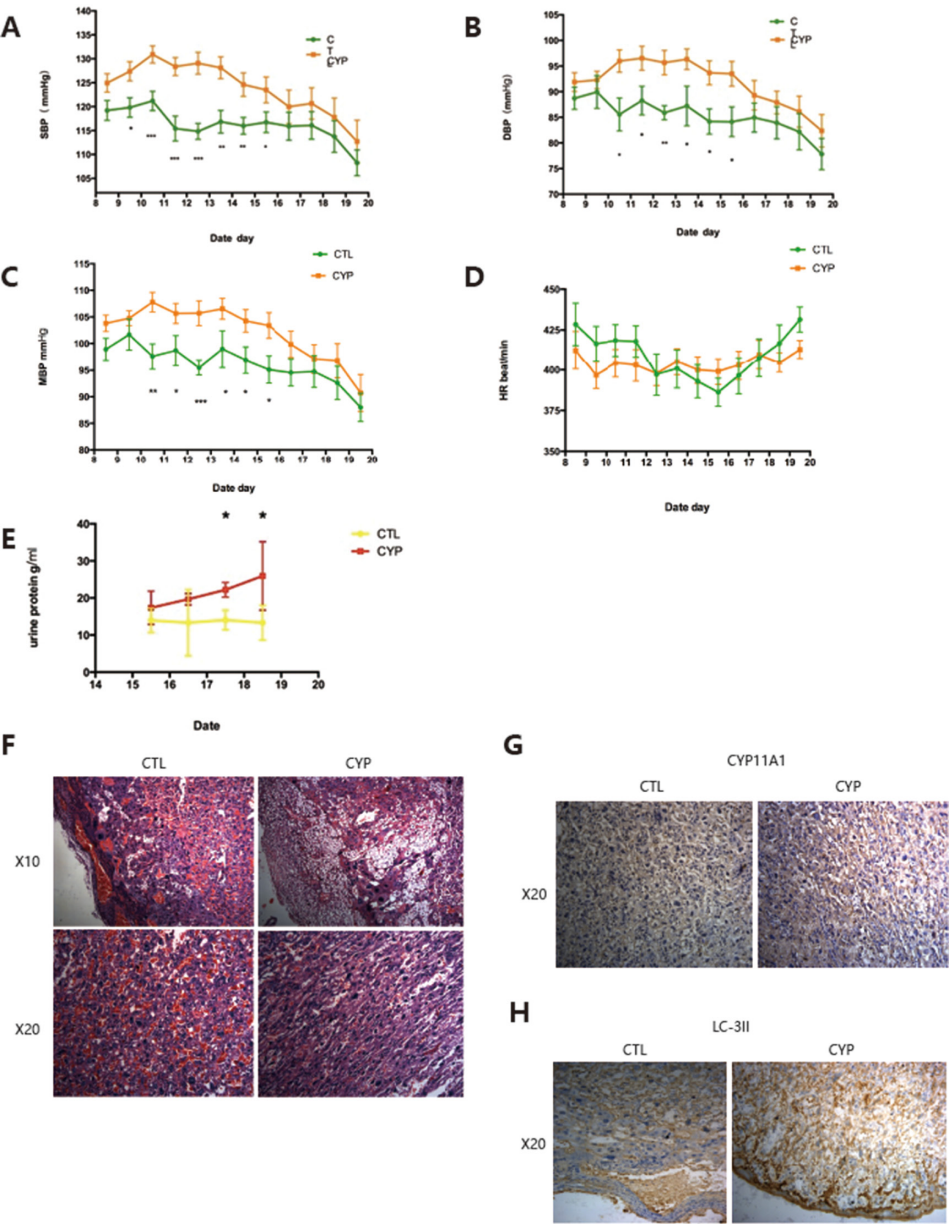
*In vivo* overexpression of CYP11A1 induce preeclampsia-like symptoms in rats (**A**) Blood pressure measurement shows that systolic pressure is significantly increased at GD 9.5 to GD 15.5 compared with control. (**B**) Diastolic pressure is significantly increased at GD 10.5 to 15.5 compared with control. (**C**) Mean blood pressure is significantly increased at GD 10.5 to 15.5 compared with control . The value is expressed as mmHg in Y axis. (**D**) Heart rate (HR) measurement shows that the HR (times/min) of CYP11A1 overexpressed rat shows no significant difference compared with control . (**E**) Urea measurement shows that higher uterine levels in overexpressed rat model. (*N* = 9) * means *P* < 0.05, ** means *P* < 0.01, *** means *P* < 0.001,****means *P* < 0.0001. (**F**) Representative images of placental histology (H&E staining) showing reduced vascularization (nucleated erythrocytes) after CYP11A1 is over-expressed in the rat model. Increased number of vacuoles cells were also evidenced in the CYP 11A1 overexpression model. Upper panel is 10 magnification (X10) and down panel is 20 magnification (X20). (**G**) Immunohistochemistry staining shows that Placental labyrinth trophoblast cells show increased expression of CYP11A1 in the animal model. X20 means 20 magnification. (**H**) Immunohistochemistry staining shows that both the decidual cells and placental labyrinth trophoblast cells show increased expression of LC-3II in the animal model. X20 means 20 magnification.

We then determined whether increased CYP11A1 expression could lead to PE-like symptoms in the rat model. Consistent with the increased level of testosterone seen, SBP, DBP, and mean blood pressure were also observed to be significantly increased in the CYP11A1 overexpression model (Figure 6A–6C), confirming that CYP11A1 overexpression can lead to increased blood pressure. Heart rate values were not changed in the CYP11A1 overexpression model (Figure 6D). Furthermore, proteinuria levels were also increased in the CYP11A1 overexpression group, indicating that CYP11A1 overexpression could cause PE-like symptoms in our rat model (Figure 6E). Hematoxylin and Eosin staining indicated that the placentas of rats overexpressing CYP11A1 showed reduced embryonic placental vascularization and altered trophoblast morphology in the placenta labyrinth, which are characteristics of PE placentas (Figure 6F). Furthermore, immunohistochemistry staining displayed an increased intensity of CYP11A1 and LC-3II in the cytoplasm of labyrinth trophoblasts in rats overexpressing CYP11A1, indicating over-activated autophagy in the placenta upon CYP11A1 overexpression *in vivo*.

Placenta weight slightly decrease but not significantly after CYP11A1 overexpression, and offspring weights showed a significant decrease after CYP11A1 overexpression (Supplementary Figure 2A–2C), indicating that CYP11A1 overexpression leads to compromised placenta development. Maternal weight gain from GD 0.5 to GD 19.5 is also significantly lower in rats overexpressing CYP11A1 compared with that in the control group (Supplemental Figure 2D). The number of resorption sites is modestly decreased while the number of viable fetuses is modestly increased in the rats overexpressing CYP11A1 compared with that in the control group (Supplemental Figure 3A). All these results indicate that CYP11A1 overexpression leads to PE-like symptoms in this rat model. Finally, we determined the expression of CYP11A1 in human PE placentas and, importantly, found that CYP11A1 expression is increased significantly in PE samples (Supplemental Figure 3B). In conclusion, this result demonstrates that CYP11A1 overexpression is a characteristic of PE patients. Our results have revealed a mechanism by which abnormally regulated CYP11A1 could activate mitophagy, thus proving to be an important etiological factor in PE pathogenesis (Supplementary Figure 3C).

## DISCUSSION

The current study aimed to investigate the detailed mechanism by which excessive CYP11A1 expression in trophoblasts could contribute to PE pathogenesis. The choriocarcinoma cell line BeWo was used for the mechanistic study, since it has previously been demonstrated that BeWo is a hormone-responsible cell line [15]. When the BeWo cells were transfected with the CYP11A1 overexpression plasmid (pcDNA-3.1-CYP11A1), levels of pregnenolone, progesterone and testosterone were all found to be significantly increased and cell invasion inhibited. This indicates that high levels of CYP11A1 may increase the levels of pregnenolone, progesterone, and testosterone, inhibiting cell invasion. In our study, the inhibition of cell invasion was rescued upon treatment with flutamide, an AR inhibitor. These results indicate that CYP11A1 overexpression may reduce trophoblast invasion by elevating testosterone levels. Other research has indicated that increased maternal testosterone, at concentrations relevant to abnormal clinical conditions, can cause hypertension associated with blunting of NO-mediated vasodilation, and that testosterone may induce the increased vascular resistance associated with pregnancy-induced hypertension [16]. The CYP11A1 gene has long been linked to the pathogenesis of polycystic ovary syndrome and the major mechanism attributed is related to the abnormal androgen metabolism [17]. Our previous study has shown that CYP11A1 gene expression is increased in PE placentas [9]. To the best of our knowledge, the current study is the first to show that prenatal exposure of CYP11A1 could lead to elevated testosterone secretion and over-activated autophagy in the placenta, which resulted in PE-like symptoms in the rat model. Together, with the CYP11A1 transgenic mice model [8], our study shows that CYP11A1 plays a critical role in trophoblast invasion. Furthermore, our results show that CYP11A1 overexpression leads to excessive testosterone secretion, over-activated autophagy, and compromised invasion; thus CYP11A1 is likely an important gene related to PE pathogenesis. It has been shown that hypomethylation of the CYP11A1 promoter could lead to increased expression of CYP11A1 in PE placentas [18]. Since androgen inhibition can rescue the inhibition of cell invasion caused by CYP11A1 overexpression, our results reveal the AR as an important target for intervention during PE pathogenesis.

The mechanism by which overproduction of CYP11A1 and testosterone leads to abnormal invasion remains unclear. Autophagy is a biological process that degrades cellular components through the lysosome pathway for survival in response to starvation [12]. Induction of autophagy provides the cell with molecular building blocks and energy. Autophagy is thought to be involved in early human placentation [19]. A number of studies have reported that typical markers of autophagy, LC3 and P62 expression, are significantly increased in placentas from pregnancies complicated by PE and intrauterine growth restriction (IUGR) [20], suggesting that autophagy plays a key role in the pathogenesis of these diseases [10, 21]. In this study, we have also confirmed increased expression of LC3 and P62 in placenta trophoblasts from PE and in BeWo cells overexpressing CYP11A1. Furthermore, the invasion of BeWo cells treated with high testosterone decreased and western blotting showed that LC3II expression was elevated. Our results further show that androgen can lead to excessive mitophagy and compromised invasion through the AR. It is possible that the AR affects autophagy through transcriptional regulation. It should be noted that whether the AR behaves as an activator or repressor of autophagy is highly tissue-specific [10, 22]. Our study provides a novel link between testosterone level, autophagy, and trophoblast invasion during early placentation.

During pregnancy, enhancement of autophagy has been found in EVTs under physiological hypoxia in early gestation, in order to achieve success in placental development [23]. However, impaired autophagy in EVTs in early placental tissue could contribute to PE pathogenesis [14]. Our data showed that CYP11A1 overexpression leads to abnormal oxidative stress and induces increased autophagy. This increased autophagy activity in trophoblasts affects trophoblast invasion. Backe *et al*., have also shown that excessive autophagy leads to reduced trophoblast invasion, which can lead to reduced MMP9 expression [24]. However, the exact mechanism by which excessive autophagy compromises trophoblast invasion is unknown. One possibility is that excessive mitophagy affects mitochondrial metabolism [25], which would affect the energy needed for normal invasion. Nevertheless, the detailed mechanism needs further investigation.

Recent research has shown that testosterone plays a critical role in pregnancy-related hypertension and could also lead to hypertension in offspring [26]. It has been shown that increased testosterone levels are observed in plasma of PE patients [7, 27]. Our study revealed a critical role of testosterone in trophoblast invasion and demonstrated that an AR inhibitor could rescue the invasion inhibition of androgen. Thus, changes in pregnancy-related hormones (such as testosterone, a previously unappreciated hormone) could be important for a pregnancy-related disease, such as PE in our study. It should be noted that CYP11A1 localizes to the mitochondria; therefore, other roles of CYP11A1 such as in cholesterol flux and mitochondrial remodeling cannot be excluded [28]. However, the development of anti-androgen therapy for hormone-related cancer [29], such as an AR inhibitor, may provide new avenues for the prevention and treatment of PE caused by abnormal regulation of hormone metabolism during pregnancy.

## MATERIALS AND METHODS

The study was approved by the Ethics Committee of Second University Hospital of Sichuan University, China. All patient-derived tissue samples were obtained with written informed consent. Consent was obtained at diagnosis from 24 women with PE. Placentas from these pregnancies as well as from 24 normotensive (delivered at term) pregnancies were collected at delivery (caesarean section), between October 2013 and December 2014, at the Department of Obstetrics and Gynecology of West China Second University Hospital, Sichuan University. PE was defined as a maternal SBP of at least 140 mmHg and/or a diastolic blood pressure of at least 90 mmHg, by measuring on two occasions at least 6 h apart. Proteinuria was defined as having greater than 300 mg during a 24-h urinary collection or qualitatively greater than 1+ in accordance with the guidelines of the American College of Obstetricians and Gynecologists. Patients with chronic medical disorders such as diabetes mellitus, cardiovascular disease, collagen disorder, chronic renal disease, chronic hypertension, or metabolic diseases were excluded. Patient characteristics, including gestational weeks, body mass index, fetal weight, blood pressure, and placenta thickness are summarized in Supplementary Table 1.

### Placental tissue preparation and histology

Placental tissue was gathered within 30 min of delivery. For western blotting, placental tissues (maternal side) were collected from midway between the edge and center of each placenta and stored at −80°C for further studies. Hematoxylin and Eosin staining were performed following the protocol of histology lab.

### Cell culture and treatments

The choriocarcinoma cell line BeWo was purchased from the American Type Culture Collection (ATCC). BeWo cells were cultured in Roswell Park Memorial Institute (RPMI) Medium 1640 basic (11875, GIBCO, USA) containing 10% fetal bovine serum (FBS, 10099, GIBCO, USA), and 1% penicillin/streptomycin. BeWo cells were divided into four groups: group 1 was transfected with a control plasmid (pcDNA-3.1), group 2 was transfected with a CYP11A1 overexpression plasmid (pcDNA-3.1-CYP11A1), group 3 was treated with 10 μM testosterone (T6147, Sigma) for 24 h, and group 4 was transfected with pcDNA-3.1-CYP11A1 and then treated with 10 μM flutamide, an androgen receptor (AR) inhibitor (F9397, sigma), for 24 h. BeWo cells were treated with 10 μM forsklin (Sigma) as a positive control for syncytiotrophoblast formation, and untreated BeWo cells were used as a basal control. Three wells were used per group for data analysis.

### Transfection

The choriocarcinoma cell line BeWo was cultured in RPMI 1640 medium supplemented with 10% FBS at 37°C in a 5% CO_2_ atmosphere. CYP11A1 cDNA was cloned from human testis cDNA and inserted into the pCDNA3.1 plasmid with BamH1 and EcoR1 restrictive enzyme sites. Lipofectamine3000 Transfection Reagent (L3000015, Invitrogen), following Invitrogen's protocol, was used to transfect the BeWo cells with pcDNA-3.1-CYP11A1. The transfection efficiency of pcDNA-3.1-CYP11A1 into BeWo cells was confirmed by western blotting; an increasing amount of plasmid DNA (500 ng, 1250 ng, 2000 ng) was transfected into BeWo cells to decide upon the optimal concentration.

### Western blotting

BeWo cells were lysed in radioimmunoprecipitation assay (RIPA) buffer (150 mM sodium chloride, 1.0% NP-40, 1% Triton X-100, 0.5% sodium deoxycholate, 0.1% sodium dodecyl sulfate (SDS), and 50 mM pH 8.0 Tris buffer). Whole cell protein was extracted by 4°C centrifugation at 12000 *g* for 30 min. Protein concentrations were determined using the Pierce BCA Protein Assay Kit (Cat. No: 23227, Thermo Scientific). Protein samples (45 μg) were loaded on 12% SDS-polyacrylamide gels, resolved by electrophoresis, and transferred to polyvinylidene difluoride (PVDF) membranes (BR07146602, 0.22 mm, Merck Millipore). Immunoblotting was performed using primary antibodies against LC3 (1:1000, 12741, CST), Beclin-1 (1:1000, 3495, CST), CYP11A1 (1:1000, 14217, CST), VDAC-1 (1:1000, Proteintech), PINK-1 (1:1000, 6946, CST), P62 (1:10000, ab109012, Abcam), MMP2 (1:2000, EPR1184, Abcam), or GAPDH (1:150000, Zen Bioscience), at 4°C overnight followed by incubation with a HRP-conjugated goat anti-rabbit IgG (1:10000, ZB-2301, ZSGB-BIO) secondary antibody at room temperature (RT) for 1 hour. The blots were exposed using a chemiluminescence kit (WBKLS0500, Merck Millipore) and the signal was detected using a gel imaging system. The corresponding internal reference GAPDH was used as an internal control. The intensity of bands was analyzed using Image J (National Institutes of Health, USA).

### Immunofluorescence

The expression intensity and pattern in BeWo cells of LC3-II, lysosome-associated membrane glycoprotein 3 (Lamp3), and E-cadherin were determined by immunofluorescence staining. After different treatments, cells, at RT, were fixed for 30 min with 4% (w/v) paraformaldehyde, permeabilized for 10 min with 0.1% Triton X-100 (T9284-500ML, Sigma Life Science), and then blocked with 1% (w/v) albumin bovine V (BS043E, Biosharp) for 1 h. The cells were incubated with the rabbit anti-human primary antibodies LC3 (1:100,12741, CST), Lamp3 (1:200, SC-6840, Santa Cruz), or E-cadherin (1:50, SC7870, Santa Cruz), and were incubated with a goat anti-rabbit IgG secondary antibody (1μg/ml, Alexa Fluor 488, A11008, Invitrogen), goat anti-mouse fluorescence in isothiocyanate-conjugated IgG (1:150, Invitrogen) and Hoechst 33342 (1:10000, Invitrogen). The nuclei were labelled by DAPI (4’,6-diamidino-2-phenylindole). Cells were imaged by confocal microscopy (Olympus FV1000, MIU-IBC). For immunostaining the mitochondria an anti-Cox-IV antibody (Zen Bioscience, Chengdu, China) was used. Four random fields in three wells per group were counted (for the figures for the migrating cells) using Image J software.

### Hormone determination using enzyme linked immunosorbent assay (ELISA) or mass spectrometry

Steroid levels were determined using ELISA. After treatments, cell supernatants were collected from 6-well plates. Pregnenolone levels were determined using the ALPCO protocol (Pregnenolone ELISA, 11-PREHU-E01), and progesterone levels were detected using radioimmunoassay in the medical clinical laboratory of West China Second University Hospital, Sichuan University. Testosterone levels were determined following the R&D protocol (Testosterone Assay, KGE010, R&D Systems). Each sample was measured in duplicates. For animal experiments blood pregnenolone levels were determined using isotope dilution high performance liquid chromatography-tandem mass spectrometry (ID-HPLC-MS/MS) as previously described, and the coefficients of variation (CV) for pregnenolone was 9.2%.

### Flow cytometry

Mitochondrial transmembrane potential (ΔΨm) was measured using the JC-1 dye with a mitochondrial membrane potential assay kit (Beyotime, Shanghai, China). After treatment, 1 × 10^6^ cells were incubated at 37°C for 20 min with 1 mL of JC-1 working solution (as recommended via the manufacturer's protocol). Then the staining solution was removed and cells were washed twice with the JC-1 staining 1x buffer. Finally, cells were resuspended in 0.6 mL of buffer and analyzed using a flow cytometer (Cytomics FC 500, Beckman Coulter) with an excitation of 490 nm and emission at 530 nm and 590 nm, respectively. ΔΨm monomers and J-aggregates were determined in triplicate using the ratio of fluorescence intensity at 530 nm to 590 nm.

### Transwell assay (invasion assay)

Invasion assays were performed using the transwell assay system; 24-well plates were used as the outer chambers and polycarbonate filters (6.5-mm Transwell with 8-μm Pores, Polyolycarbonate Membrane Insert, Sterile, 3422, CORNING) were used as the inner chambers. The upper surface of the inner chamber was coated with 30 μl of diluted matrigel (1:9 in RPMI-1640, 356234, Becton Dickinson Company, USA). After treatments, 200 μl of BeWo cell suspension (2.0 ×10^5^ cells) was seeded into the inner chamber. The outer chamber contained 600 μl of complete culture medium (RPMI-1640 containing 30% FBS). Cells in the chamber were cultured for 24 h and the cells on the filter were methanol-fixed for 15 min. Non-migrated cells on the upper surface of the filter were removed by gently scraping with a cotton swab. Migrated cells on the lower surface of the filter were stained using crystal violet and imaged using a Nikon ECLIPSE Ti microscope (Japan). Migrated cells were counted in 10 random fields using the Image J software.

### Animal model

Virgin female Sprague–Dawley rats were purchased from Chengdu Dashuo Experimental Animal Inc. Virgin female Sprague–Dawley rats (240–260 g) were housed in a light- and humidity-controlled environment and fed freely with access to water. In the morning, after females were housed over-night with a fertile male (280–310 g) at a 1:1 ratio, vaginal plugs were observed. The presence of vaginal plugs confirmed pregnancy and was designated gestational day 0.5 (GD 0.5). Timed-pregnant rats were divided into 2 groups with each group containing 10 pregnant rats on GD 7.5, and received different injections on GD 8.5. Group 1 received injections into their tail veins of 1×10^9^ plaque formation units (PFUs) of CYP11A1 gene containing adenovirus (AD-CYP11A1, 15879-1, GENECHEN), while group 2 received the control vehicle adenovirus (ADCON177, GENECHEN). Both groups had their blood pressures measured from GD 8.5 before injection to GD 19.5. The blood pressures were measured using a rat's noninvasive sphygmomanometer (BP-2010A, Beijing Softron Biotechnology) and the average of three different trials over a 20-min period was recorded. Rats were trained prior to blood pressure monitoring from GD 5.5 to GD 7.5 to adapt to the rat's noninvasive sphygmomanometer. Blood, tissue and fetal matter were obtained after anesthesia (10% w/v chloralic hydras, intraperitoneal injection) on GD 19.5. Plasma pregnenolone was measured by mass spectrometry, while progesterone and testosterone were measured by radioimmunoassay. Levels of CYP11A1 expression and autophagy markers in the placenta were confirmed by western blotting. A single voided urine sample was collected from GD 15.5 to GD 19.5 after the blood pressure test, and urinary protein was measured using the Pierce BCA Protein Assay Kit (23227, Thermo Scientific).

### Statistical analysis

Data are presented in this study as mean ± standard deviation (S.D.). The GraphPad Prism software package (version 6.0; La Jolla, California, USA) was used for statistical analysis. The *t*-test or Mann–Whitney *U* test was performed to compare two groups. The one-way ANOVA test was used to assess differences among more than two groups. The Newman-Keuls post-hoc multiple comparisons test was used to determine the difference between each two groups. The variances in data from BeWo cells were shown via S.D. and the animal models’ variance were displayed in the graphs using standard error. A probability of *P* < 0.05 was considered statistically significant.

## SUPPLEMENTARY MATERIALS FIGURES AND TABLES


