# Supplementary material for: Abnormal CYP11A1 gene expression induces excessive autophagy, contributing to the pathogenesis of preeclampsia
Source: Oncotarget. 2017 Sep 22;8(52):89824–36. doi: 10.18632/oncotarget.21158 (PMC5685712; doi:10.18632/oncotarget.21158)
Supplement: Supplementary file 1 [file oncotarget-08-89824-s001.pdf]

Abnormal CYP11A1 gene expression induces excessive autophagy, contributing to the pathogenesis of preeclampsia

SUPPLEMENTARY MATERIALS

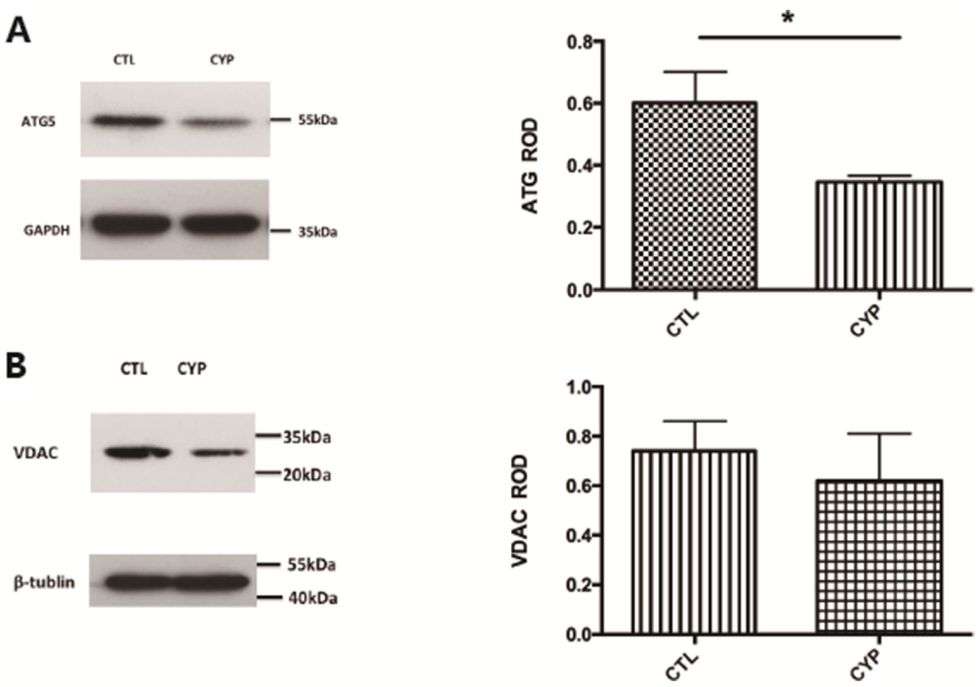

Supplementary Figure 1: (**A**, **B**) Western blot shows that ATG5 and VDAC1 expression are suppressed in CYP11A1 overexpression BeWo cells. * means $P < 0.05$.

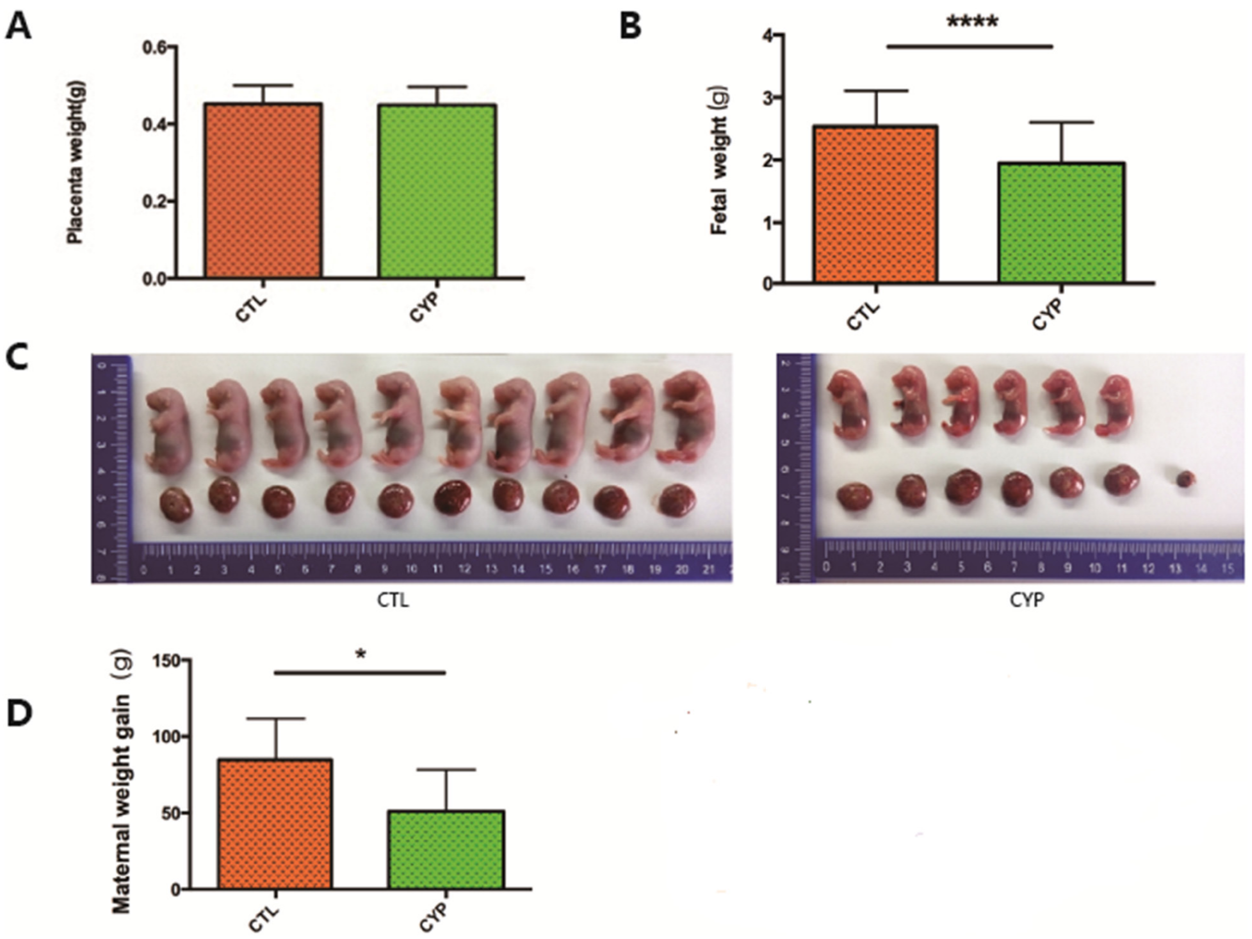

Supplementary Figure 2: *In vivo* overexpression of CYP11A1 leads to decreased placenta, fetal weight and reduced maternal weight gain in rats. (**A**) Placenta weight shows significant decrease after overexpression of CYP11A1. (**B**, **C**) Offspring weights shows significant decrease after overexpression of CYP11A1. (**D**) Overexpression of CYP11A1 Rats' maternal weight again from GD 0.5 to GD 19.5 is significant lower than the control group. * means $P < 0.05$, ** means $P < 0.01$, *** means $P < 0.001$,****means $P < 0.0001$.

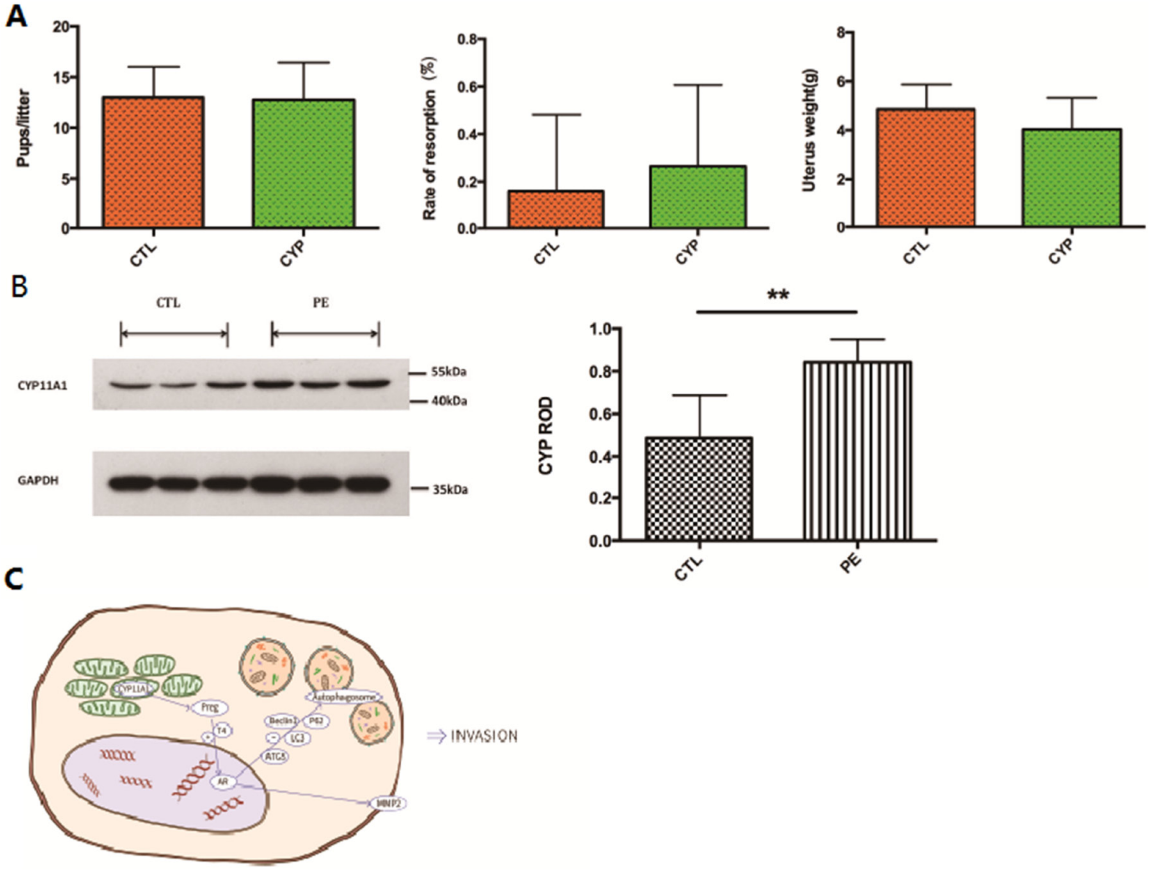

Supplementary Figure 3: (**A**) Pregnancy loss and fetal number shows modest decrease after overexpression of CYP11A. (**B**) Western blot result shows that the expression of CYP11A1 is significantly increased in placenta of preecampsia patients (*N* = 24 for control group; *N* = 24 for PE group); (**C**) The diagram shows the proposed mechanism of how overexpressed CYP11A1 leading to compromised trophoblast invasion, therefore contributing preeclampsia pathogenesis. Over-activated CYP11A1 increases androgen production, which stimulate androgen receptor (AR) transcriprional activity. Androgen receptor (AR) could then activate the expression of mitophagy related proteins, including LC-3, Beclin 1, P62, et, al. The excessive mitophagy inhibits the trophoblast invasion through regulating MMP2 expression.

Supplementary Table 1: Clinical charactericstics of patients in the current study

	**Healthy pregnant (*n* = 24)**	**Preeclampsia (*n* = 24)**	***P* value**	***P* significant**
Age (years old)	30.02 ± 0.6790	32.34 ± 1.209	0.1021	ns
gestational age (weeks) at end of pregnant	39.79 ± 0.1836	34.98 ± 0.9715	< 0.0001	****
number of parity	1.417 ± 0.1797	2.792 ± 0.2885	0.0002	***
namber of gestation	1.083 ± 0.05763	1.292 ± 0.1123	0.0723	ns
fetal weight (g)	3358 ± 61.45	2264 ± 263.5	0.0005	***
fetal length (cm)	49.79 ± 0.2169	43.45 ± 1.724	0.0016	**
placenta weight (g)	601.2 ± 21.77	431.3 ± 34.57	0.0001	***
BMI at beginning of pregnant (kg/m^2)	20.25 ± 0.4121	22.49 ± 0.8989	0.0403	*
BMI at deliver (kg/m^2)	25.44 ± 0.3993	28.08 ± 0.8163	0.0071	**
SPB (mm/Hg)	110.4 ± 1.340	149.2 ± 3.468	< 0.0001	****
DPB (mm/Hg)	67.59 ± 2.460	95.45 ± 2.230	< 0.0001	****
